# Effects of physical training on physical and functional fitness, physical activity level, endothelial function, hemodynamic variables, bone metabolism, and quality of life of post-bariatric patients: study protocol for a randomized controlled trial

**DOI:** 10.1186/s13063-022-06677-z

**Published:** 2022-09-02

**Authors:** Karynne Grutter Lopes, Maria das Graças Coelho de Souza, Michelle da Costa Tavares Bezerra, Lucas Miranda Bessa, Paulo Farinatti, Eliete Bouskela, Miguel Madeira, Luiz Guilherme Kraemer-Aguiar

**Affiliations:** 1 Postgraduate Program in Clinical and Experimental Physiopathology, Faculty of Medical Sciences, State University of Rio de Janeiro, Rio de Janeiro, RJ Brazil; 2 Obesity Unit, Centro de Pesquisa Clínica Multiusuário (CePeM), Hospital Universitário Pedro Ernesto (HUPE), State University of Rio de Janeiro, Rio de Janeiro, RJ Brazil; 3 Laboratory of Clinical and Experimental Research in Vascular Biology (BIOVASC), State University of Rio de Janeiro, Rio de Janeiro, RJ Brazil; 4 Federal University of Rio de Janeiro - Endocrinology Division, Rio de Janeiro, RJ Brazil; 5 Laboratory of Physical Activity and Health Promotion, Institute of Physical Education and Sports, University of Rio de Janeiro State, Rio de Janeiro, RJ Brazil; 6 Postgraduate Program in Exercise and Sports Sciences, University of Rio de Janeiro State, Rio de Janeiro, RJ Brazil; 7 Endocrinology, Department of Internal Medicine, Faculty of Medical Sciences, State University of Rio de Janeiro, Rio de Janeiro, RJ Brazil

**Keywords:** Obesity, Bariatric surgery, Muscle mass, Muscle strength, Bone health, Randomized controlled trials

## Abstract

**Background:**

Evidence of the benefits induced from resistance exercise on health markers of post-bariatric patients is limited. The study will investigate the effects of a resistance training (RT) program on muscle mass and strength, bone metabolism biomarkers, bone mineral density (BMD), bone microarchitecture, and endothelial function of patients subjected to Roux-en-Y gastric bypass.

**Methods/design:**

This randomized controlled trial will include 60 post-bariatric patients, physically inactive, aging 18 to 50 years, with a post-surgery period ≥ 12 months. They will be randomly assigned into two groups: (i) the non-exercised control group, which will receive the standard clinical follow-up, or (ii) the intervention group which will consist of RT (60 min/session; 3 times/week, for 6 months). The primary outcomes will include muscle mass and strength, bone metabolism biomarkers, BMD, and bone microarchitecture. The secondary outcomes will be anthropometry, hemodynamic measurements, cardiovascular risk factors, health-related quality of life (QoL), and endothelial function. Outcomes will be assessed by blood biomarkers of bone formation and reabsorption, dual X-ray absorptiometry, repetition maximum and handgrip strength tests, high-resolution peripheral quantitative computed tomography, 36-Item Short-Form Health Survey, venous occlusion plethysmography, and nailfold videocapillaroscopy.

**Discussion:**

It is expected that there are greater benefits from the RT program, possibly improving muscle mass and strength, bone metabolism, density and microarchitecture, QoL, and cardiovascular risk.

**Trial registration:**

ClinicalTrials.gov NCT04193397. Registered on 7 December 2019.

**Supplementary Information:**

The online version contains supplementary material available at 10.1186/s13063-022-06677-z.

## Background

Obesity is a chronic disease associated with multiple comorbidities resulting from the interaction of genetic, metabolic, environmental, and behavioral factors [1]. The prevalence of excess body weight has increased alarmingly over the last few decades. Globally, at least 2.8 million people die from overweight or obesity-related illnesses each year, putting obesity as a significant public health problem [2, 3]. On the other hand, bariatric surgery is an effective therapy to promote significant weight loss compared to clinical treatment, reducing total mortality and improvement or remiting type 2 diabetes, hyperlipidemia, hypertension, and obstructive sleep apnea [4–6].

Despite several clinical benefits, such as reductions in postoperative adverse events and mortality rates, some complications may occur following surgery [7, 8], due to a parallel loss of fat-free mass. Therefore, the bones and muscles may be in part negatively affected.

Unfortunately, there is accumulated evidence demonstrating a reduction in bone mineral density (BMD) [9, 10] after a bariatric procedure. Studies that compared the two most commonly performed techniques [11] showed significantly more bone loss after Roux-en-Y gastric bypass (RYGB) than after sleeve gastrectomy (SG), especially in the lumbar spine, total hip, and femoral neck [9, 10]. This undesired event may result in greater skeletal frailty and risk of fractures [12, 13]. Deleterious effects of bariatric surgery on bone metabolism are related to decreased mechanical stress, calcium and vitamin D malabsorption, secondary hyperparathyroidism, deficiencies of macro- and micro-nutrients, and changes in fat mass and gut hormone secretion [14, 15].

Postoperative muscle loss [16, 17] leads to decreased physical function and resting energy expenditure that contributes to regain of weight [18] and, partly, to relapse of obesity-related comorbidities [6]. It is already known that the advance of age is significantly involved in the loss of muscle mass and strength. Sarcopenia may result from both conditions, leading to difficulties in daily life activities, loss of autonomy, and quality of life (QoL), with higher morbidity and mortality [19].

Strategies to attenuate bone frailty and muscle atrophy in post-bariatric patients are needed, and a resistance training (RT) program can induce positive adaptations in the musculoskeletal system [20, 21], as well as in the stimulus of osteogenic response with preserved BMD in clinical studies [22–24]. In addition, RT contributes to preventing several cardiometabolic risk factors and is considered an independent predictor of all-cause mortality in the general population [25]. However, evidence of the benefits of RT on muscle and bone health in post-bariatric patients is limited. In these patients, additional studies with larger sample sizes, longer exercise interventions, higher volume, and progression overload exercises, and assessment of clinical parameters are necessary to assess the potential of RT on bone health and on fat-free mass integrity [26].

Thus, the present study will investigate the effects of an RT program on muscle mass and strength and on bone metabolism biomarkers, mineral density, and microarchitecture, and endothelial function of patients subjected to RYGB. We hypothesized that the RT program would lead to more remarkable improvement in the assessed outcomes than standard recommendations, as well as in the cardiovascular risk factors and health-related QoL.

## Methods

### Study design

This is a randomized controlled trial, parallel-group, superiority trial with a 1:1 allocation ratio for two arms of treatment. This study protocol will be conducted at the Centro de Pesquisa Clínica Multiusuário (CePeM), Laboratory of Physical Activity and Health Promotion (LABSAU), Laboratory of Clinical and Experimental Research in Vascular Biology (BIOVASC) from the State University of Rio de Janeiro (UERJ), and at the Hospital Universitário Clementino Fraga Filho (HUCFF) from Federal University of Rio de Janeiro (UFRJ) (RJ, Brazil). Patients who were subjected to a RYGB will be recruited at our outpatients’ care unit.

**Fig. 1:**
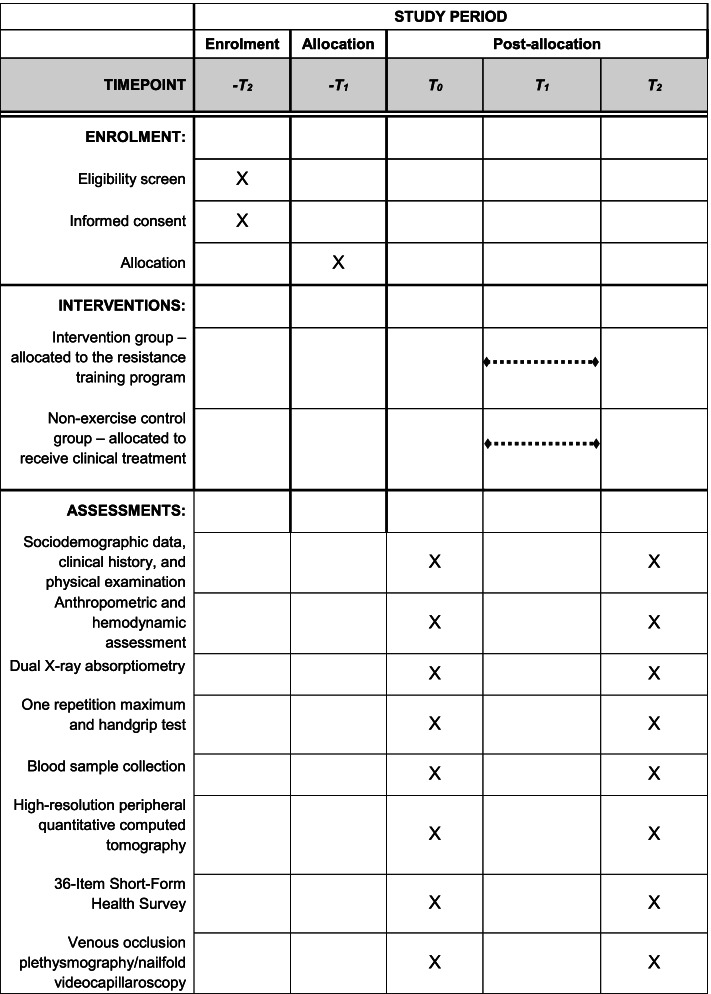
Schedule of enrollment, interventions, and assessments. −t2, enrolment week; −t1, allocation; t0, pre-intervention; t1, 6 months of intervention; t2, post-intervention

Recruitment, pre-participation screening, allocation, intervention, and data collection will occur between October 2022 and December 2023. We plan that the study will be concluded, and the results will be available for analysis in December 2024. The present study protocol complies with the SPIRIT 2013 recommendations (Standard Protocol Items: Recommendations for International Trials) [27] (see Additional file 1). Figure 1 shows the schedule of enrollment, interventions, and assessments.

### Recruitment and eligibility criteria

We will approach patients subjected exclusively to RYGB in regular clinical follow-up, aging 18 to 50 years and after at least 12 months from surgery. Our outpatients’ care unit follows at least 180 patients subjected to a bariatric procedure each year. Considering the inclusion and exclusion criteria, we expect to recruit 40 to 60 patients. The exclusion criteria will include regular physical exercise (≥ 3 days/week during 30 min) in the last 6 months; smoking; alcoholism; pregnancy; diagnosis or evidence of any cardiovascular; respiratory, neurological, infectious, endocrine, or musculoskeletal impairments that limit physical exercise; and or being in the use of any hormonal replacement therapy or medications that influence bone metabolism. Those who start some physical exercise program during the study, or with excess weight loss (EWL) < 50% (considered as primary non-responders) [28], or being in the use of drugs that interfere with weight, or who have performed a revisional bariatric surgery will also be excluded.

### Randomization and experimental design

Sixty post-bariatric patients will be randomized through a random code generator software (www.randomization.com) at a ratio of 1:1 into two groups: (a) intervention group—that will perform a supervised RT program for 6 months—or (b) non-exercised control group—that will receive the standard clinical follow-up and shall not change their physical activity behavior. All patients will be followed by a multidisciplinary team (compose of endocrinologists, nutritionists, and physical educators) with expertise in post-bariatric patients, following the clinical practice guidelines [29]. Once the study is completed, for ethical reasons, the exercised control group will be invited to carry out the same RT program. Usual clinical care will be maintained for all patients following completion of the trial at our outpatients’ care unit.

**Fig. 2:**
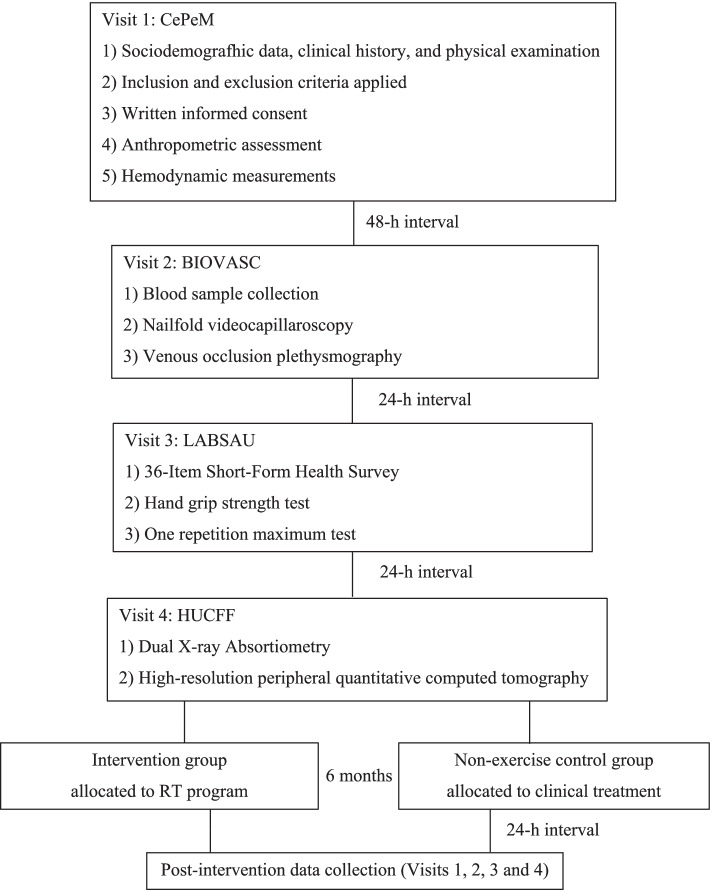
Experimental design. CePeM, Centro de Pesquisa Clínica Multiusuário; LABSAU, Laboratory of Physical Activity and Health Promotion; BIOVASC, Laboratory of Clinical and Experimental Research in Vascular Biology; HUCFF, Hospital Universitário Clementino Fraga Filho

The participants will visit our lab four times for data collection before and after 6 months post-intervention, with at least 24- to 48-h intervals between visits, as shown in Fig. 2. All assessments will be held in the morning between 7 and 11:00 am, in a quiet temperature-controlled environment (21–23 °C). The participants will be instructed to fast for 8 h for blood sample collections and vascular measures (at visit 2). The primary outcomes will be muscle mass and strength, bone metabolism biomarkers, BMD, and bone microarchitecture. The secondary outcomes will be anthropometry, hemodynamic measurement, metabolic/cardiovascular risk factors, health-related QoL, and endothelial function.

### Intervention

Patients in the intervention arm will participate in an RT program for 6 months. The exercise sessions will consist of (a) 10-min specific warm-up, consisting of one set of 20 repetitions with loads corresponding to 50% of the resistance used in the first exercise of the training session; (b) 45-min of resistance exercises for the major muscle groups of the upper and lower body (8 single and multi-joint exercises with free weights and machines, including seated rowing, leg press, bench press, leg flexion, pull down, leg extension, shoulder press, and abdominal exercises), performed with 2–3 sets of 8–12 repetitions with loads ≥ 70% of one-repetition maximum (1RM) interspersed with 2 min intervals between sets; and (c) 5-min recovery with stretch and cool-down exercises. The OMNI-res scale will be used after each set of resistance exercises to rank patients’ perceived exertion [30]. All training sessions will occur indoors (21–23 °C), three times a week on nonconsecutive days, from 7 to 10 am, and under the supervision of the research staff. The patients will be instructed on the importance of the benefits of regular RT to improve adherence to the intervention.

### Primary outcomes measures

#### Dual X-ray absorptiometry (DXA)

Body composition will be assessed using a Prodigy-GE densitometer (GE Lunar Prodigy Advance, GE Healthcare Madison, WI, USA). Appendicular skeletal muscle mass (ASM) will be calculated with the results obtained. BMD at the lumbar spine (L1–L4), femoral neck, total femur, and one-third distal radius will be expressed in absolute values (g/cm^2^) and as standard deviations (SD) from the expected BMD for the age-matched population. A *Z*-score ≤ − 2.0 SD is considered a low bone mass for age, whereas a *Z*-score above 2.0 is within the expected range for the period, according to the 2019 Official Positions of the International Society for Clinical Densitometry. All DXA measurements will be performed by the same experienced technologist [31].

#### One repetition maximum (1RM) test

The dynamic strength maximal assessed by the 1RM test will be used to determine the loads applied during the RT program. After the general warm-up of 5 min, the volunteers will perform a specific warm-up set of 8 repetitions at approximately 50% of the estimated 1RM, followed by another set of 3 repetitions at 70% of the estimated 1RM. The patients will be verbally encouraged to perform 3 to 5 attempts for each exercise with a rest interval of 5 min between them. An interval of 10 min will be allowed before the start of the test for the next exercise. The 1RM will be determined as the higher load achieved.

#### Handgrip strength test and sarcopenia cutoff points

Isometric maximal handgrip strength will be measured by hand dynamometer TSD121C Hand Dynamometer (Biopac Systems, Inc., CA, USA) with the patients seated in a chair, with elbows flexed and the forearm in supinated position (dominant arm). The patients will perform three attempts of 5 s interspersed with 2-min intervals, and the highest value will be recorded as a result.

Sarcopenia will be detected by cutoff points of low muscle strength and low muscle mass, assessed by the handgrip test and ASM measurement, respectively, defining it as probable or confirmed sarcopenia, according to current consensus [19]. Sarcopenia cutoff points for low muscle strength from Dodds et al. [32] and low muscle mass from Baumgartner et al. [33] will be applied, considering the ratio between ASM by DXA and height squared (ASM index) of 5.45 and 7.26 kg/m^2^ and a handgrip strength of 16 and 27 kg for women and men, respectively.

#### Biochemical parameters

##### Methodology for hemogram, glycemia, lipid profile, and blood total protein evaluations

Hemogram will be performed by automatically counting blood cells from whole blood samples. Plasma glucose levels will be determined by the colorimetric glucose oxidase method. Blood will also be collected to assess the serum insulin levels and lipid profile. Serum insulinemia will be evaluated by an electrochemiluminescent immunoassay (Elecsys Insulin Assay, Roche Diagnostics GmbH, Mannheim, Germany) through an automatic analyzer (Cobas e411; Roche Diagnostics, Munich, Germany), as previously described [34]. The serum levels of triglycerides (TG), total cholesterol (TC), high-density lipoprotein cholesterol (HDL-c), and total proteins will be assessed by colorimetric methods glycerol-phosphate oxidase/peroxidase, cholesterol oxidase/peroxidase, direct detergent, and biuret, respectively. All analyses will be performed using commercially available kits suitable for an A25 automatic analyzer (BioSystems, Barcelona, Spain). Low-density lipoprotein cholesterol (LDL-c) will be calculated using the Friedwald equation [35].

##### Methodology for determinations of calcium, phosphorus, and total alkaline phosphatase blood concentrations

Analysis of serum levels of calcium, phosphorus, and total proteins will be performed by colorimetric methods purple phthalein, phosphorus molybdate, and Modified Roy [36], respectively. All analyzes will be performed using commercially available kits from Latest (Lagoa Santa, Minas Gerais, Brazil) using an automatic analyzer.

##### Methodology for determination of blood levels or activity of bone metabolism biomarkers using enzyme immunoassays (ELISA)

In this study, we will determine the following: blood levels of amino-terminal propeptide of type 1 procollagen (P1NP), osteocalcin, and blood activity of bone alkaline phosphatase (BAP), which are related to bone formation. In addition, we will also assess the blood levels of the soluble ligand of nuclear factor kappa B receptor activator (sRANKL) and degradation products of C type I collagen telopeptide (CTX-1), both related to bone resorption. We will also measure parathyroid hormone (PTH) and vitamin D blood levels [37–39].

Blood levels of sRANKL will be determined by sRANKL HS ELISA technique (Biomedica Medizinprodukte GmbH, Vienna, Austria) [40]. In this assay, the soluble RANKL present in the samples binds to the recombinant human osteoprotegerin that coats the microplate wells. Then, the sRANKL already bound to osteoprotegerin is recognized by a goat polyclonal anti-sRANKL biotinylated detection antibody. Horseradish peroxidase (HRP)-conjugated streptavidin is added to wells and binds to the sRANKL biotinylated detection antibody. The solution containing the substrate of the enzyme peroxidase (hydrogen peroxide) and chromogen (tetramethylbenzidine (TMB)) is added to the microplate. At this stage, the peroxidase present in the sample wells reacts with hydrogen peroxide, and the reaction product oxidizes TMB, changing the color of the solution to blue. The intensity of the color is directly proportional to the concentration of the conjugated enzyme and, consequently, of the sRANKL.

Blood levels of vitamin D and P1NP will be evaluated by vitamin D (Calbiotech, El Cajon, CA, USA) [41] and P1NP ELISA Kit (MyBioSource, San Diego, CA, USA) [42], respectively. In brief, these assays are based on the competitive interaction between biotin-labeled vitamin D and P1NP and those unlabeled vitamin D and P1NP (present in standards or samples) which bind to antibodies specific to vitamin D and P1NP that coat the microplate wells. After incubation, the unbound vitamin D and P1NP are washed off, and then, avidin conjugated to HRP is added to each microplate well and incubated. The amount of bound HRP conjugate and color intensity developed after substrate solution addition are inversely proportional to the amount of vitamin D and PINP present in the samples.

Osteocalcin will be measured using the Osteocalcin EIA kit (DRG International, Inc., Springfield, NJ, USA) [43]. This sandwich format ELISA kit utilizes two antibodies: one immobilized on the microplate wells and a biotynilated one, which is incubated with standards and samples. After a washing cycle, a second incubation with a streptavidin-HRP conjugate is performed. After the addition of substrate solution, which contains hydrogen peroxide and TMB, the HRP activity is determined by means of the color intensity developed which is directly proportional to the amount of osteocalcin present in standards and sample wells.

The serum levels of PTH will be determined by the PTH ELISA kit (DRG International, Inc., Springfield, NJ, USA) [43]. In this assay, antibodies raised against two specific regions on the PTH molecule are present. One of them is biotinylated while the other is HRP-labeled. Both form the sandwich complex necessary for PTH detection. The biotinylated antibody present in the sandwich complex binds to streptavidin that coats the microwells. Thus, standards and samples are simultaneously incubated with biotin- and HRP-labeled antibodies in streptavidin-coated wells. After the incubation period, the microplate wells are washed to remove unspecific bindings, and the TMB-containing substrate solution is added. The color intensity developed is directly proportional to the PTH levels in the samples.

Serum crosslaps (CTX-1) (Immunodiagnostic Systems, IDS, Boldon Colliery, UK) [44] will be used to determine the serum and plasma concentrations of human CTX-1 degradation products. This sandwich ELISA assay is based on two highly specific monoclonal antibodies against the amino acid sequence of EKAHD-ß-GGR. In order to detect specifically the CTX degradation products, the two chains of EKAHD-ß-GGR should be cross-linked. The two monoclonal antibodies, one biotinylated and the other HRP-conjugated, are simultaneously incubated with standards and samples on microtiter wells pre-coated with streptavidin.

Since the complex between the cross-linked EKAHD-ß-GGR and biotin- and HRP-labeled antibodies are formed, it binds to the microplate immobilized streptavidin by means of the biotinylated antibody. After this incubation, the wells are washed and the chromogenic substrate is added, and the observed optical density is directly proportional to the levels of CTX-1 degradation products.

BAP blood activity will be evaluated using the Quidel MicroVue BAP kit (Quidel, San Diego, CA, USA). This assay consists, firstly, of the capture of BAP present in the samples by a monoclonal antibody anti-BAP. After capture, the enzymatic chromogenic substrate, p-nitrophenol phosphate, is added to each well, and the color intensity will be read spectrophotometrically, which is proportional to the BAP activity [45].

All procedures will be performed according to the instructions provided by the kits’ manufacturers, and the optical density of each of the 96-wells of the microplate will be measured using an Universal Microplate Reader (Bio Tek model ELX 800, Winooski, VT, USA).

The levels or activity of bone metabolism markers will be determined by correlating the optical density value of the samples with the optical density values of the standard curve with the aid of an adequate data analysis program (KC Junior, Bio Tek, Winooski, VT, USA).

#### High-resolution peripheral quantitative computed tomography (HR-pQCT)

Volumetric BMD and bone microarchitecture will be measured on the non-dominant distal radius and tibia using a three-dimensional HR-pQCT system (XtremeCT, Scanco Medical AG, Brüttisellen, Switzerland), as previously described [46]. The variables that will be analyzed include the following: (i) volumetric BMD for total, trabecular, and cortical regions (Tt.vBMD, Tb.vBMD, and Ct.vBMD; mg of hydroxyapatite/cm^3^); (ii) cortical thickness, calculated by dividing the cortical volume by the external bone surface (CtTh; mm); (iii) percentage of trabecular bone volume, i.e., ratio of BV to total volume (BV/TV; %); (iv) trabecular thickness (TbTh; mm); (v) trabecular number (TbN; 1/mm); (vi) trabecular separation (TbSp; mm); and (vii) SD of TbSp (TbSp 1/N SD; mm).

### Secondary outcomes measures

#### Anthropometry measurements

An electronic scale and stadiometer (Welmy™ W300A, São Paulo, SP, Brazil) will be used to measure body mass and height. Body mass index (BMI) will be calculated as the weight, in kilograms, divided by the squared height, in meters. Neck, waist, and hip circumferences will be measured by flexible measuring tape according to the standard procedures, and waist-to-height ratio will be calculated.

Presurgery weight and minimum postsurgery weight will be self-reported by patients [47]. EWL and rate of weight regain (RWR) will be calculated as follows: (i) EWL = (presurgery weight − minimum postsurgery weight)/(presurgery weight − ideal weight for BMI of 25 kg/m^2^) × 100% and (ii) RWR = (current weight − minimum weight postsurgery)/(presurgery weight − minimum weight postsurgery) × 100% [48].

#### Hemodynamic and cardiometabolic risk factors measurements

Resting blood pressure and heart rate will be assessed in the non-dominant arm by a semi-automated oscillometric device (G-TechTM BSP11, Hangzhou, Zhejiang, China) in a controlled and quiet environment after 10-min resting in a sitting position, according to the standard recommendations [49]. Cardiometabolic risk factors will be evaluated by BMI, waist circumference, blood pressure, triglycerides, HDL-c, LDL-c, total cholesterol, and fasting plasma glucose [50].

#### 36-Item Short-Form Health Survey (SF-36)

Health-related QoL will be assessed using the SF-36 [51]. This tool consists of scores varying from 0 to 100 in eight health domains (physical function, physical role, pain, general health, vitality, social functioning, emotional role, and mental health). A higher score reflects better health perception. Those who grade 50 points or less will indicate poorer health than the reference population [52].

#### Venous occlusion plethysmography and nailfold videocapillaroscopy

Vascular function (reflected by endothelial-dependent and independent vasodilation) will be evaluated by venous occlusion plethysmography (HokansonTM AI6, Bellevue, WA, USA). There will 4 stages as follows: (1) baseline forearm blood flow (FBF) 1, (2) FBF during reactive hyperemia (after 5-min forearm arterial occlusion with pressure 50 mmHg above SBP), (3) baseline FBF 2, and (4) FBF after 5 min of 0.4 mg sublingual nitroglycerin (Nitrolingual BurnsAdler Pharmaceuticals™, Charlotte, NC, USA). FBF will be measured for 2 min at each stage with 3 min intervals for each phase will be performed, except between reactive hyperemia and baseline FBF 2, which will 15 min for washout of vasodilating substances created during ischemia [53].

Microvascular function and morphology will be evaluated through nailfold videocapillaroscopy. The capillary diameters (afferent, apical, and efferent densities), functional capillary density (i.e., number of capillaries with flowing red blood cells), baseline red blood cell velocity (RBCV), maximal RBCV (after 1 min of arterial blood flow occlusion), and time to achieve RBCVmax will be measured [53].

### Sample size calculation and statistical analysis

In this study, it will be used a similar sample size previously used in a trial which provides 90% detection power between-group differences of BMD, with a significance level of 0.05 [54]. Descriptive analysis will consist of means and SD for continuous variables and percentages for categorical variables. Data normality will be tested by the Shapiro-Wilk test with the results expressed as mean ± SD or percentage, whenever appropriate. Baseline variables will be compared between the groups using either the Student *t*-test or chi-squared test. To compare dependent variables, a two-way ANOVA will be used considering group (intervention and non-exercised control groups) and time points (pre and post-intervention), followed by the Bonferroni post hoc test, in case of significant *F* ratios. An intention-to-treat analysis will be conducted, i.e., all participants, regardless of adherence to the intervention, will be included in the final analysis. No additional analyses are planned for the study. All calculations will be performed using the GraphPad™ software (version 6.0, La Jolla, CA, USA), and the significance level will be set at *P* ≤ 0.05.

### Withdrawals of study and data/safety monitoring plan

The criteria for discontinuation will be (i) participant request, (ii) changes of clinical status during exercise sessions, (iii) absence ≥ 80% of training sessions, and (iv) engagement in another physical exercise program during the study. The principal investigator will oversee and monitor all elements of the trial, with contributions from co-investigators as needed. Patients who present any clinical adverse event during RT sessions will be conducted to the emergency room at our university hospital. Adverse clinical events will be reported to the ethics committee. The Data Monitoring Committee will consist of the principal investigator and co-investigators, who will perform meetings each month throughout the trial period to review data reports on recruitment, screening, baseline and post-interventions assessments, protocol adherence, adverse events, and safety. Since no anticipated problems which could be detrimental to the volunteers are expected in this trial, no interim analysis will be performed.

### Data collection, management, and dissemination

Checklists will be developed to guarantee the quality and completeness of the data and procedures. In order to keep confidentiality, all participants’ data will be coded with study ID and stored in folders, checked for completeness by the principal investigator, and will not be passed on to any third party without consent from participants. Study copy documentation will be stored securely at the university with restricted access, during and after the completion of the study and after data publication. Each participant will receive a full report containing his/her assessment results and information about physical exercise recommendations at the end of the study [55]. The results of the trial will be publicized in scientific congresses and journals.

### Masking and blinding

In this study, it will not be possible to blind either patients or investigators who will administer the RT program. On the other hand, a blind researcher will perform the randomization of patients into the study groups. An evaluator and statistician will perform data collection, outcome assessors, and analysis blinded to group allocation.

### Plans for collection, laboratory evaluation, and storage of biological specimens for genetic or molecular analysis in the trial/future use

Blood samples will be collected through the venous puncture and analyzed in our laboratories. All biological specimens will be destroyed after data is recorded. No specimens will be retained for future use.

## Discussion

Physical exercise programs could positively influence bone and muscle health in several clinical populations [21–24, 56, 57], including post-bariatric patients [26]. This benefit is relevant, given the relationship between osteoporosis and sarcopenia vs. disability and mortality [19, 58]. On the other hand, available evidence suggests that several surgical results are worsened by lack of physical exercise leading to the return of obesity-related comorbidities and regression in QoL [47, 59]. In this context, defining which exercise strategy better suits this population and which one is more effective in improving these outcomes is relevant.

Previous studies have demonstrated this prospect, but the assessment of all these outcomes is needed in a large randomized controlled trial [60–62]. In summary, the effects of RT on clinical and health consequences of post-bariatric patients are not consensual, probably due to the methodological limitations of the current literature. In general, trials that applied isolated RT programs used various experimental designs, including training variables (frequency, intensity, duration, volume, or progression), the sample used (small sample size, only females or both sexes, or participants of different ages and clinical conditions), the nutritional status (supplementation use or not), and also the start period of the RT program after surgery [26]. Moreover, even considering RT the most effective exercise used to preserve skeletal integrity [54], data on the literature concerning the influence of an isolated RT program on bone adaptations and bone turnover in post-bariatric patients is scarce.

RT will be prescribed in the present protocol. As already known, RT is a non-pharmacological strategy widely used for clinical care and physical performance. This type of exercise is an essential component of any physical training program [21]. A prolonged RT program performed with high mechanical loading promotes an appropriate stimulus that improves the musculoskeletal system in post-bariatric patients without compromising safety. This improvement was previously demonstrated in postmenopausal women [23], HIV-infected individuals [22], and subjects with cardiovascular diseases [25, 63], cancer [57, 63], and renal diseases [24]. Thus, bone density and microarchitecture measurements, bone formation/resorption markers, endocrine responses, body composition, muscle mass, and strength will be performed, which may help the explanation of our findings. In addition, for those with obesity, the prescription of RT has effectively reduced cardiovascular risk factors, controlled body weight, and improved physical and mental health, indicating the clinical importance of applying this training method to these patients [64–66]. Obesity and its metabolic effects have a complex and, unfortunately, relapsing nature. Therefore, another critical and original aspect of our study refers to the measures of cardiovascular risk, surgery data, and QoL altogether since we could not locate prior studies comparing the assessment of all these outcomes post-RT vs. clinical treatment [26].

The present protocol has some limitations. Firstly, although all patients will be required to record their daily routines and be encouraged to maintain sleep hours and healthy eating habits during the study, we cannot ensure that it will occur. Moreover, it will not be possible to assess the patients before surgery and include other types of bariatric procedures. These would be useful to observe the magnitude of changes in clinical parameters at pre- and post-surgery periods and also between surgical techniques. Even taking care of these limitations, the present study might extend the current knowledge in this area. RT will possibly promote cardiometabolic benefits while preventing adverse effects of bariatric surgery on bone and muscle health. Our future data may help clarify the potential application of prescribing this training method as a clinical tool in managing post-bariatric patients.

## Trial status

It is not yet recruiting. Start date: October 2022. Expected completion date: December 2024.

## Supplementary Information

**Additional file 1:** SPIRIT checklist.

**Additional file 2:** Ethics committee approval letter.

## Data Availability

The dataset analyzed will be available from the corresponding author upon reasonable request and approval from the applicable Institutional Review Board.

## Abbreviations

ASM: Appendicular skeletal muscle mass
BAP: Bone alkaline phosphatase
BMD: Bone mineral density
BP: Blood pressure
BV/TV: Percentage of trabecular bone volume
P1NP: Amino-terminal propeptide of type 1 procollagen
CtTh: Cortical thickness
CTX-1: C type I collagen telopeptide
DXA: Dual X-ray absorptiometry
ELISA: Enzyme-linked immunosorbent assay
EWL: Excess weight loss
FBF: Forearm blood flow
HDL-c: High-density lipoprotein cholesterol
HRP: Horseradish peroxidase
HRR: Heart rate reserve
LDL-c: Low-density lipoprotein cholesterol
QoL: Quality of life
RM: Repetition maximum
RYGB: Roux-en-Y gastric bypass
RWR: Rate of weight regain
SD: Standard deviation
SF-36: 36-Item Short-Form Health Survey
SPIRIT: Standard Protocol Items: Recommendations for International Trials
sRANKL: Soluble ligand of nuclear factor kappa B receptor activator
P1NP: Amino-terminal propeptide of type 1 procollagen
PTH: Parathyroid hormone
RBCV: Red blood cell velocity
TBM: Tetramethylbenzidine
TbN: Trabecular number
TbSp: Trabecular separation
TbSp 1/N SD: Standard deviation of trabecular separation
TbTh: Trabecular thickness
TC: Total cholesterol
TG: Triglycerides
