# Supplementary material for: Effects of physical training on physical and functional fitness, physical activity level, endothelial function, hemodynamic variables, bone metabolism, and quality of life of post-bariatric patients: study protocol for a randomized controlled trial
Source: Trials. 2022 Sep 2;23:733. doi: 10.1186/s13063-022-06677-z (PMC9438121; doi:10.1186/s13063-022-06677-z)
Supplement: Supplementary file 2 — Additional file 2: Ethics committee approval letter. [file 13063_2022_6677_MOESM2_ESM.pdf]

PARECER CONSUBSTANCIADO DO CEP

DADOS DO PROJETO DE PESQUISA

Título da Pesquisa: EFEITOS DO TREINAMENTO FÍSICO SOBRE A APTIDÃO FÍSICO-FUNCIONAL, NÍVEL DE ATIVIDADE FÍSICA, FUNÇÃO ENDOTELIAL, VARIÁVEIS HEMODINÂMICAS, METABOLISMO ÓSSEO E QUALIDADE DE VIDA DE PACIENTES SUBMETIDOS À CIRURGIA BARIÁTRICA: UM ESTUDO CONTROLADO

Pesquisador: Luiz Guilherme Kraemer de Aguiar

Área Temática:

Versão: 1

CAAE: 16425419.8.0000.5259

Instituição Proponente: Hospital Universitário Pedro Ernesto/UERJ

Patrocinador Principal: Financiamento Próprio

DADOS DO PARECER

Número do Parecer: 3.461.120

Apresentação do Projeto:

Transcrição editada do conteúdo do registro do protocolo e dos arquivos anexados à Plataforma Brasil.

Objetivo: Estudar os efeitos de um programa de treinamento concorrente sobre a aptidão físico-funcional, nível de atividade física, função endotelial,

pressão arterial, marcadores bioquímicos de risco cardiovascular e do metabolismo ósseo, densidade e microestrutura ósseas e indicadores de

qualidade de vida de pacientes que realizaram cirurgia bariátrica (Estudo 1). Comparar as possíveis mudanças ósseas e musculares em pacientes

que foram submetidos à cirurgia bariátrica através da técnica da gastroplastia redutora com reconstrução em Y de Roux (GRYR) com controles não

bariátricos, assim como, correlacionar esses indicadores de saúde com o tempo decorrido do procedimento cirúrgico e a perda ponderal (Estudo 2).

Métodos: O ensaio clínico controlado e randomizado (Estudo 1) contará com pacientes que foram submetidos à cirurgia bariátrica, em atendimento

ambulatorial na Policlínica Piquet Carneiro (PPC). Após o recrutamento, os pacientes serão randomizados em dois grupos: treinamento (GT) e

Endereço: Avenida 28 de Setembro 77 - Térreo
Bairro: Vila Isabel **CEP:** 20.551-030
UF: RJ **Município:** RIO DE JANEIRO
Telefone: (21)2868-8253 **E-mail:** cep.hupe.interno@gmail.com

controle (GC). Os pacientes alocados em GT realizarão, durante seis meses, um programa de exercícios aeróbios e resistidos, com duração
aproximada de 60 minutos. As sessões terão frequência de três vezes por semana, incluindo: 20 minutos de caminhada em esteira rolante, com
intensidade correspondente a 20% acima da velocidade de conforto e 40 minutos de exercícios de força envolvendo grandes grupamentos
musculares, com 8–12 repetições em carga correspondente a 70–85% de 1 repetição máxima (RM). Os pacientes em GC não modificarão suas
rotinas, sendo-lhes vedada a realização de atividades físicas sistemáticas durante o período do experimento. Ao final de três e seis meses (36 e 72
sessões, respectivamente), os desfechos serão avaliados por: a) composição corporal: densitometria com emissão de raios-X de dupla energia
(DXA); b) densidade mineral óssea volumétrica (DMOv) e microarquitetura óssea: tomografia computadorizada quantitativa periférica de alta
resolução (HR-pQCT) c) aptidão físico-funcional: teste de 1 RM e teste cardiopulmonar de exercício máximo (TCPE) d) variáveis bioquímicas: perfil
lipídico sanguíneo, glicemia e biomarcadores de formação e absorção óssea e metabolismo do cálcio (sangue e urina); e) função endotelial,
morfologia e função microvascular: ultrassom doppler vascular e videocapilaroscopia do leito periungueal; f) pressão arterial: medida casual em
consultório e monitorização ambulatorial (MAPA); g) nível de atividade física: acelerometria (actiGraph GT3X+) e International Physical Activity
Questionnaire (IPAQ) e h) indicadores de qualidade de vida: 36-Item Short-form Health Survey (SF-36). O estudo transversal (Estudo 2) contará com
dois grupos: cirurgia bariátrica (GB) – pacientes que foram submetidas à GRYR e grupo controle (GC) – controles não bariátricos pareados por sexo,
idade, cor e IMC com o GB. Os desfechos serão avaliados por: a) composição corporal: densitometria com emissão de raios-X de dupla energia
(DXA); b) DMOv e microarquitetura óssea: HR-pQCT; c) força muscular: força de preensão manual; d) variáveis bioquímicas: perfil lipídico, glicemia
e biomarcadores de formação e absorção óssea e metabolismo do cálcio (sangue e urina).Palavras-chave: obesidade; cirurgia metabólica;
treinamento concorrente; risco cardiovascular; saúde.

Objetivo da Pesquisa:

Transcrição editada do conteúdo do registro do protocolo e dos arquivos anexados à Plataforma Brasil.

Estudar os efeitos de um programa de treinamento concorrente na aptidão físico-funcional, nível de atividade física, função endotelial, pressão
arterial, marcadores bioquímicos de risco cardiovascular e do metabolismo ósseo, densidade e microestrutura ósseas e indicadores de qualidade de
vida de pacientes pós bariátricos (Estudo 1). Adicionalmente, pretende-se comparar as possíveis mudanças ósseas e musculares em pacientes que
foram submetidos à cirurgia bariátrica através da técnica da GRYR com controles não bariátricos, assim como, correlacionar esses indicadores de
saúde com o tempo decorrido do procedimento cirúrgico e a perda ponderal (Estudo 2).

Avaliação dos Riscos e Benefícios:

Transcrição editada do conteúdo do registro do protocolo e dos arquivos anexados à Plataforma Brasil.

Riscos: Informamos a não existência de riscos conhecidos.
Benefícios: Estudo 1: Promoção de efeitos benéficos na composição corporal, força muscular, aptidão cardiorrespiratória, perfil lipídico e glicemia,
biomarcadores de formação e reabsorção óssea e do metabolismo do cálcio, densidade mineral e microarquitetura óssea, função endotelial e
pressão arterial de pacientes pós-bariátricos que participaram do programa de treinamento concorrente, assim como, aumento dos níveis de
atividade física habitual e melhora de indicadores de qualidade de vida desses indivíduos;
Estudo 2: Comparar as possíveis mudanças ósseas e musculares em pacientes que foram submetidos à cirurgia bariátrica através da técnica da
GRYR com controles não bariátricos e correlacionar esses indicadores de saúde com o tempo decorrido do procedimento cirúrgico e a perda
ponderal de curto e longo prazo poderá elucidar os potenciais efeitos deletérios dessa forma de intervenção para o tratamento da obesidade grave e
ajudar no manejo adequado desses pacientes.

Prezado pesquisador: Caracteriza-se como risco direto para os participantes da pesquisa a possibilidade de desconforto ou constrangimento no momento do preenchimento dos questionários. Os pesquisadores devem se comprometer a minimizar os riscos ou desconfortos que possam vir a ser causados.

Comentários e Considerações sobre a Pesquisa:

Transcrição editada do conteúdo do registro do protocolo e dos arquivos anexados à Plataforma Brasil.

Um programa de treinamento físico com duração seis meses, incluindo exercícios aeróbios e de força muscular (treinamento concorrente) com
intensidade leve a moderada, consistirá em intervenção apropriada para promover efeitos benéficos na composição corporal, força muscular, aptidão
cardiorrespiratória, perfil lipídico e glicemia, biomarcadores plasmáticos de formação e reabsorção óssea e no osteometabolismo, função endotelial
e pressão arterial de pacientes que realizaram cirurgia bariátrica, assim como, aumentar o seu nível geral de atividade física habitual e melhorar sua
qualidade de vida (Estudo 1).
Pacientes que foram submetidos à cirurgia bariátrica, através da técnica da gastroplastia redutora com reconstrução em Y de Roux, apresentam
reduções da massa óssea com predomínio de elevação dos biomarcadores de reabsorção óssea sobre os de formação, da massa magra e da força
muscular (Estudo 2)

A pesquisa está bem estruturada e o referencial teórico e metodológico estão explicitados, demonstrando aprofundamento e conhecimento necessários para sua realização. As referências estão adequadas e a pesquisa é exequível.

Considerações sobre os Termos de apresentação obrigatória:

Foram analisados os seguintes documentos de apresentação obrigatória:

1) Folha de Rosto para pesquisa envolvendo seres humanos: Documento devidamente preenchido, datado e assinado
2) Projeto de Pesquisa: Adequado
3) Orçamento financeiro e fontes de financiamento: adequado/apresentado
4) Termo de Consentimento Livre e Esclarecido ou Justificativa: Adequado
5) Cronograma: pertinente as informações
6) Documentos pertinentes à inclusão do HUPE: Adequado
7) Currículo do pesquisador principal e demais colaboradores: anexados e conforme as normas.

Os documentos de apresentação obrigatória foram enviados a este Comitê, estando dentro das boas práticas e apresentando todos dados necessários para apreciação ética e tendo sido avaliadas as informações contidas na Plataforma Brasil e as mesmas se encontram dentro das normas vigentes e sem riscos iminentes aos participantes envolvidos de pesquisa.

Recomendações:

Os TCLE's devem se chamados de "Termo de Consentimento Livre e esclarecido" ou invés "FORMULÁRIO DE INFORMAÇÃO AO PACIENTE".

Conclusões ou Pendências e Lista de Inadequações:

O projeto pode ser realizado da forma como está apresentado. Diante do exposto e à luz da Resolução CNS nº466/2012, o projeto pode ser enquadrado na categoria – APROVADO.

Considerações Finais a critério do CEP:

Em consonância com a resolução CNS 466/12 e a Norma Operacional CNS 001/13, o CEP recomenda ao Pesquisador: Comunicar toda e qualquer alteração do projeto e no termo de consentimento livre e esclarecido, para análise das mudanças; Informar imediatamente qualquer evento adverso ocorrido durante o desenvolvimento da pesquisa; O Comitê de Ética solicita a V. Sª., que encaminhe relatórios parciais de andamento a cada 06 (seis) Meses da pesquisa e ao término, encaminhe a esta comissão um sumário dos resultados do projeto; Os dados individuais de todas as etapas da pesquisa devem ser mantidos em local seguro por 5 anos para possível auditoria dos órgãos competentes.

Este parecer foi elaborado baseado nos documentos abaixo relacionados:

Tipo Documento	Arquivo	Postagem	Autor	Situação
Informações Básicas do Projeto	PB_INFORMAÇÕES_BÁSICAS_DO_PROJETO_1347105.pdf	06/05/2019 17:35:25		Aceito
Folha de Rosto	FOLHA_DE_ROSTO.pdf	06/05/2019 17:32:26	Luiz Guilherme Kraemer de Aguiar	Aceito
TCLE / Termos de Assentimento / Justificativa de Ausência	TCLE_ESTUDO_1.pdf	05/05/2019 11:27:54	Luiz Guilherme Kraemer de Aguiar	Aceito
TCLE / Termos de Assentimento / Justificativa de Ausência	TCLE_ESTUDO_2.pdf	05/05/2019 09:56:12	Luiz Guilherme Kraemer de Aguiar	Aceito
Projeto Detalhado / Brochura Investigador	PROJETO_DETALHADO.pdf	04/05/2019 17:27:40	Luiz Guilherme Kraemer de Aguiar	Aceito
Cronograma	CRONOGRAMA.pdf	04/05/2019 17:27:23	Luiz Guilherme Kraemer de Aguiar	Aceito
Declaração de Instituição e Infraestrutura	DECLARACAO_DE_CIENCIA.pdf	01/05/2019 09:52:09	Luiz Guilherme Kraemer de Aguiar	Aceito

Situação do Parecer:
Aprovado

Necessita Apreciação da CONEP:
Não

RIO DE JANEIRO, 18 de Julho de 2019

Assinado por:
WILLE OIGMAN
(Coordenador(a))

PARECER CONSUBSTANCIADO DO CEP

DADOS DA EMENDA

Título da Pesquisa: EFEITOS DO TREINAMENTO FÍSICO SOBRE A APTIDÃO FÍSICO-FUNCIONAL, NÍVEL DE ATIVIDADE FÍSICA, FUNÇÃO ENDOTELIAL, VARIÁVEIS HEMODINÂMICAS, METABOLISMO ÓSSEO E QUALIDADE DE VIDA DE PACIENTES SUBMETIDOS À CIRURGIA BARIÁTRICA: UM ESTUDO CONTROLADO

Pesquisador: Luiz Guilherme Kraemer de Aguiar

Área Temática:

Versão: 2

CAAE: 16425419.8.0000.5259

Instituição Proponente: Hospital Universitário Pedro Ernesto/UERJ

Patrocinador Principal: Financiamento Próprio

DADOS DO PARECER

Número do Parecer: 3.631.095

Apresentação do Projeto:

Emenda para aprovação de documentação e alteração de informações relativas ao protocolo.

Objetivo da Pesquisa:

Emenda para aprovação de documentação e alteração de informações relativas ao protocolo.

Avaliação dos Riscos e Benefícios:

Emenda para aprovação de documentação e alteração de informações relativas ao protocolo.

Comentários e Considerações sobre a Pesquisa:

Justificativa da Emenda:

O projeto intitulado "EFEITOS DO TREINAMENTO FÍSICO SOBRE A APTIDÃO FÍSICO-FUNCIONAL, NÍVEL DE ATIVIDADE FÍSICA, FUNÇÃO ENDOTELIAL, VARIÁVEIS HEMODINÂMICAS, METABOLISMO ÓSSEO E QUALIDADE DE VIDA DE PACIENTES SUBMETIDOS À CIRURGIA BARIÁTRICA: UM ESTUDO CONTROLADO" foi submetido à Plataforma Brasil sob o CAAE número 16425419.8.0000.5259

para o CEP do Hospital Universitário Pedro Ernesto - UERJ, tendo sido aprovado pelo esse comitê. Entretanto, temos nesse projeto um co-participante, o prof. Miguel Madeira, do Hospital Universitário Clementino Fraga Filho - UFRJ. Esse projeto foi enviado ao IPUB - Instituto de Psiquiatria da UFRJ e consequentemente devidamente devolvido. Peço que enviem esse projeto (CAAE acima) para a Instituição co-participante HUCFF-UFRJ cujo CEP é o de número 5257.

Considerações sobre os Termos de apresentação obrigatória:

Os documentos de apresentação obrigatória foram enviados a este Comitê, estando dentro das boas práticas e apresentando todos dados necessários para apreciação ética e tendo sido avaliadas as informações contidas na Plataforma Brasil e as mesmas se encontram dentro das normas vigentes e sem riscos iminentes aos participantes envolvidos de pesquisa.

Conclusões ou Pendências e Lista de Inadequações:

A emenda apresenta todas as informações necessárias para avaliação ética. Diante do exposto e à luz da Resolução CNS nº466/2012, a Emenda pode ser enquadrada na categoria – APROVADO.

Considerações Finais a critério do CEP:

Em consonância com a resolução CNS 466/12 e a Norma Operacional CNS 001/13, o CEP recomenda ao Pesquisador: Comunicar toda e qualquer alteração do projeto e no termo de consentimento livre e esclarecido, para análise das mudanças; Informar imediatamente qualquer evento adverso ocorrido durante o desenvolvimento da pesquisa; O Comitê de Ética solicita a V. Sª., que encaminhe relatórios parciais de andamento a cada 06 (seis) Meses da pesquisa e ao término, encaminhe a esta comissão um sumário dos resultados do projeto; Os dados individuais de todas as etapas da pesquisa devem ser mantidos em local seguro por 5 anos para possível auditoria dos órgãos competentes.

Este parecer foi elaborado baseado nos documentos abaixo relacionados:

Tipo Documento	Arquivo	Postagem	Autor	Situação
Informações Básicas do Projeto	PB_INFORMAÇÕES_BÁSICAS_1400453_E1.pdf	07/08/2019 10:27:22		Aceito
Folha de Rosto	FOLHA_DE_ROSTO.pdf	06/05/2019 17:32:26	Luiz Guilherme Kraemer de Aguiar	Aceito

TCLE / Termos de Assentimento / Justificativa de Ausência	TCLE_ESTUDO_1.pdf	05/05/2019 11:27:54	Luiz Guilherme Kraemer de Aguiar	Aceito
TCLE / Termos de Assentimento / Justificativa de Ausência	TCLE_ESTUDO_2.pdf	05/05/2019 09:56:12	Luiz Guilherme Kraemer de Aguiar	Aceito
Projeto Detalhado / Brochura Investigador	PROJETO_DETALHADO.pdf	04/05/2019 17:27:40	Luiz Guilherme Kraemer de Aguiar	Aceito
Cronograma	CRONOGRAMA.pdf	04/05/2019 17:27:23	Luiz Guilherme Kraemer de Aguiar	Aceito
Declaração de Instituição e Infraestrutura	DECLARACAO_DE_CIENCIA.pdf	01/05/2019 09:52:09	Luiz Guilherme Kraemer de Aguiar	Aceito

Situação do Parecer:

Aprovado

Necessita Apreciação da CONEP:

Não

RIO DE JANEIRO, 09 de Outubro de 2019

Assinado por:

WILLE OIGMAN

(Coordenador(a))
